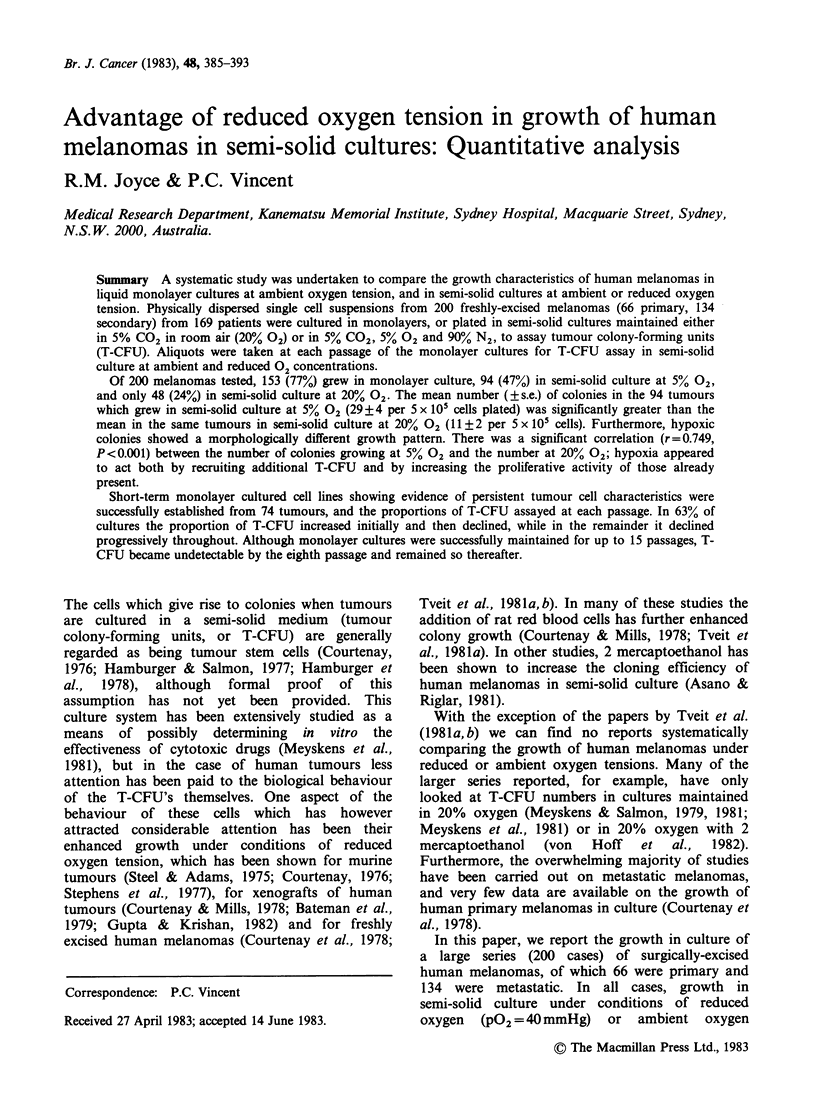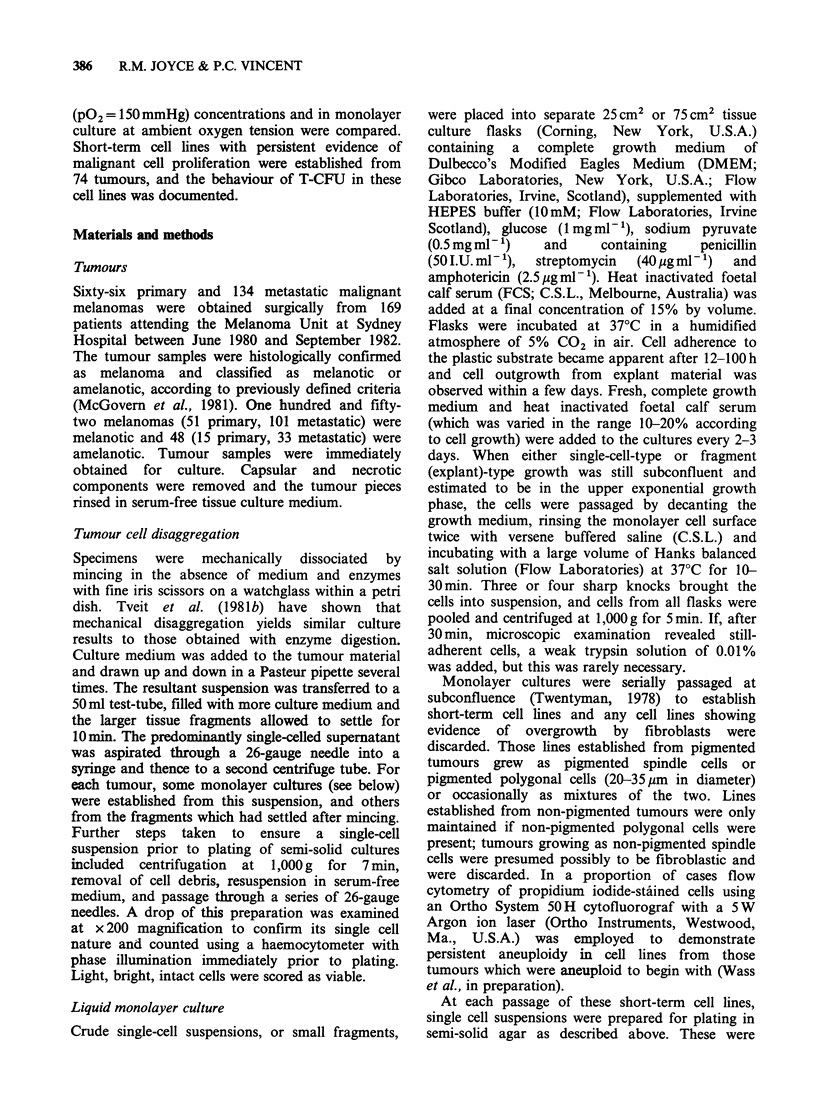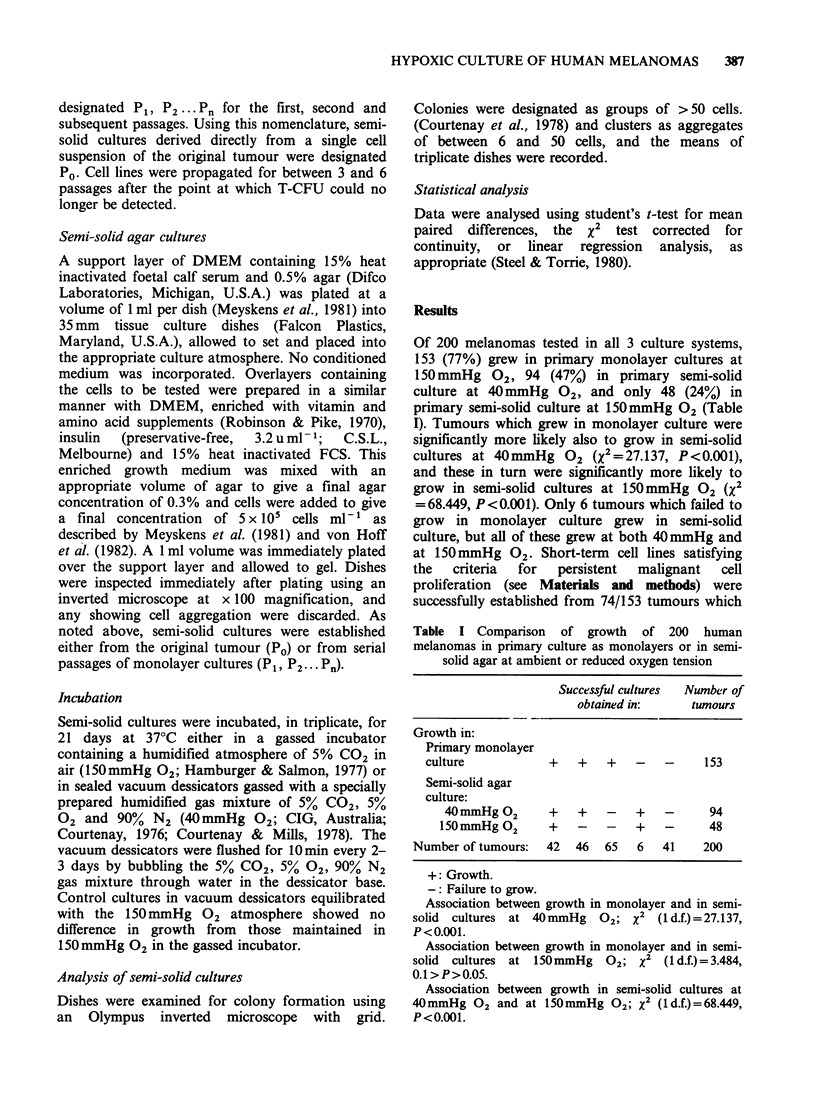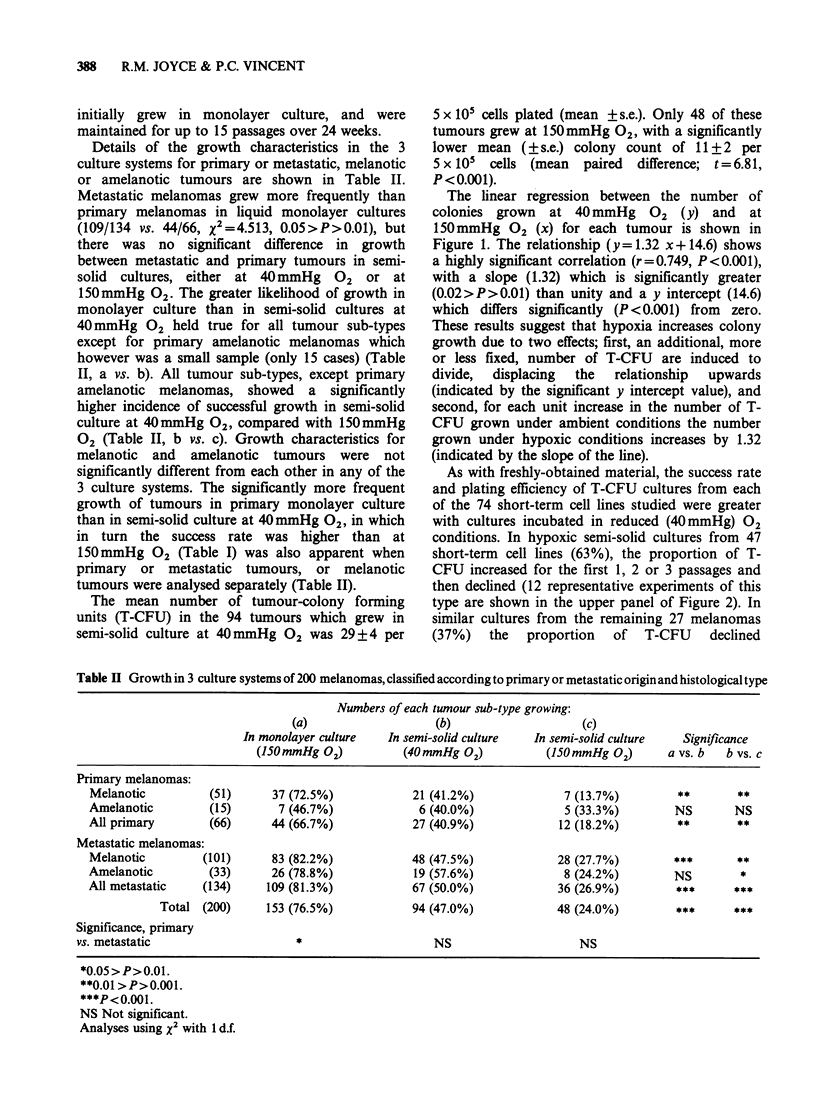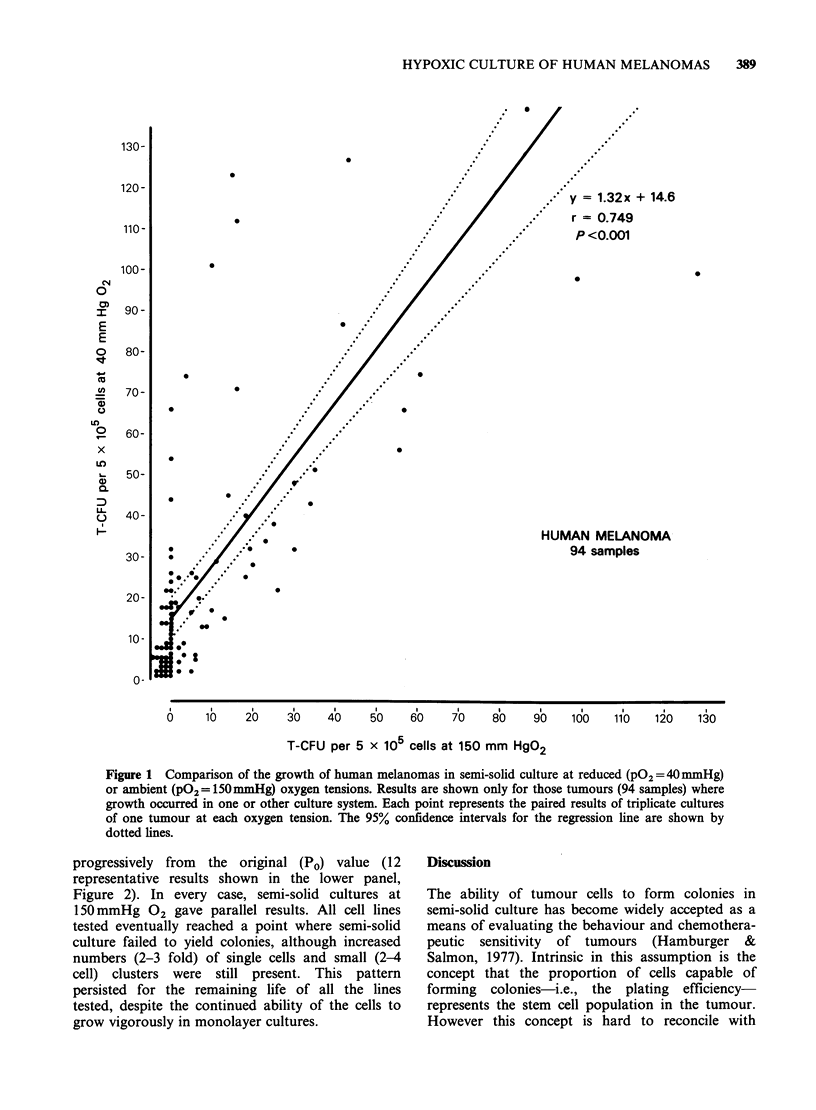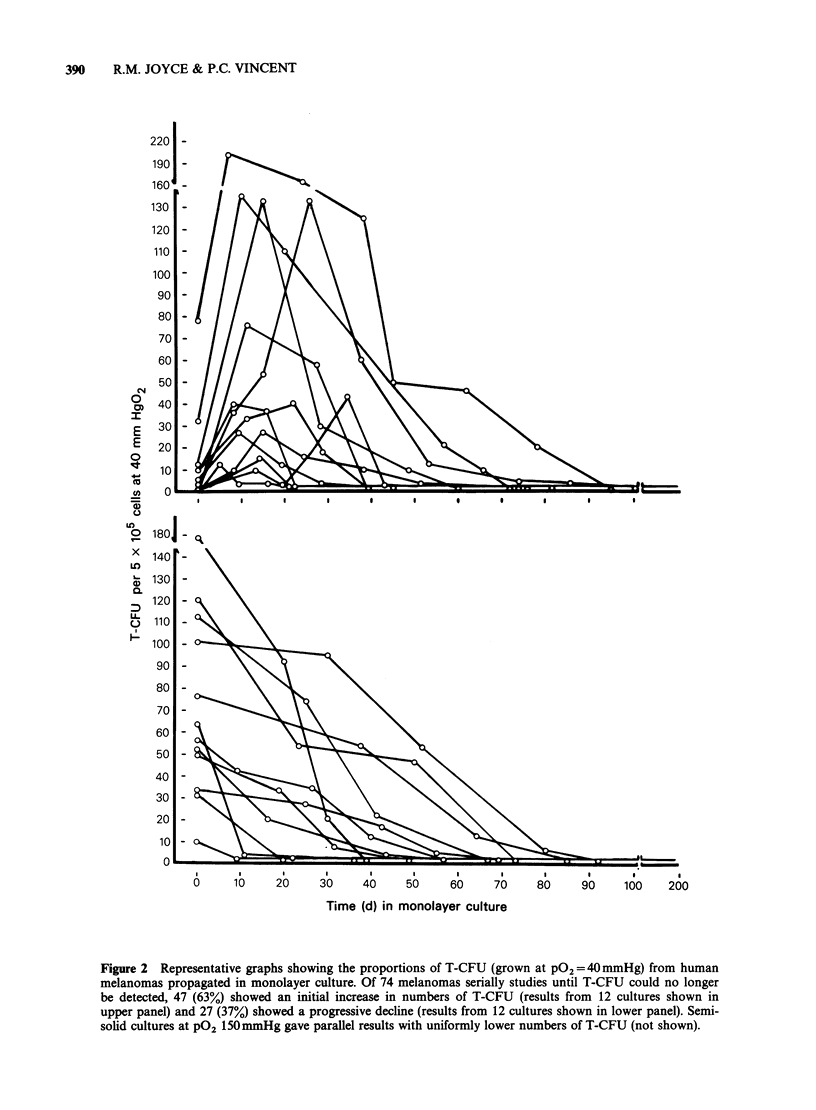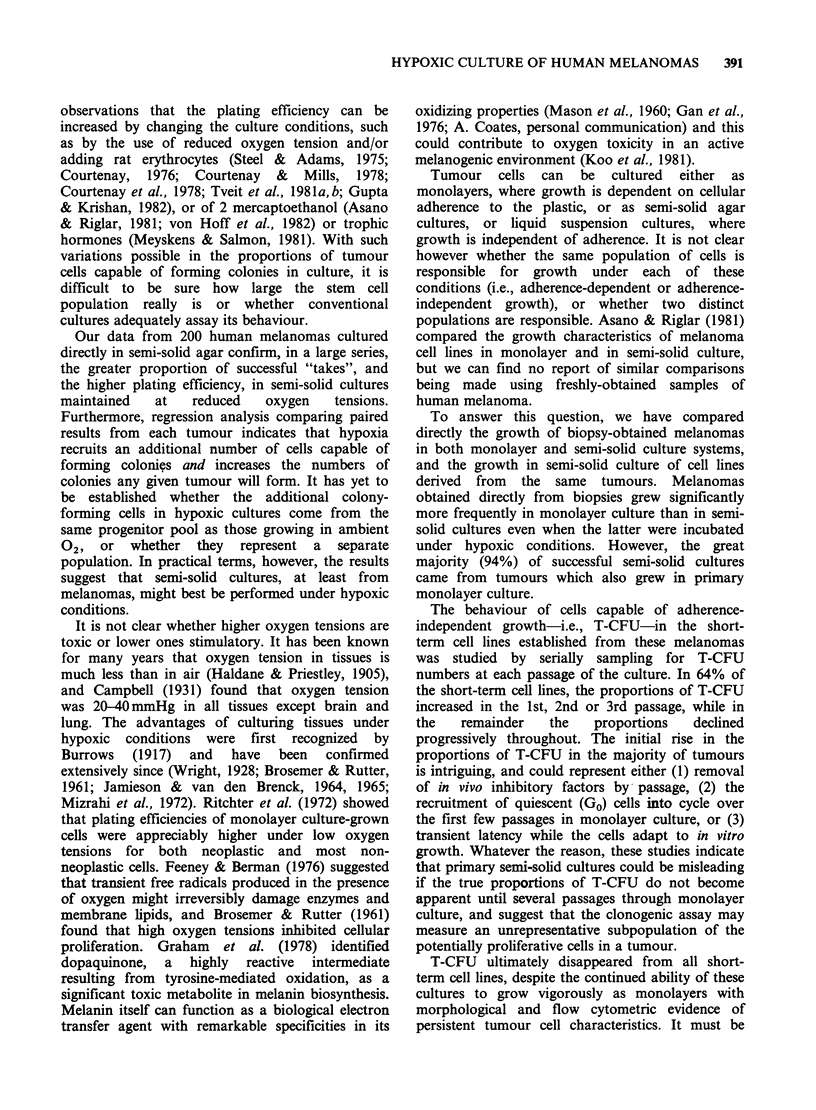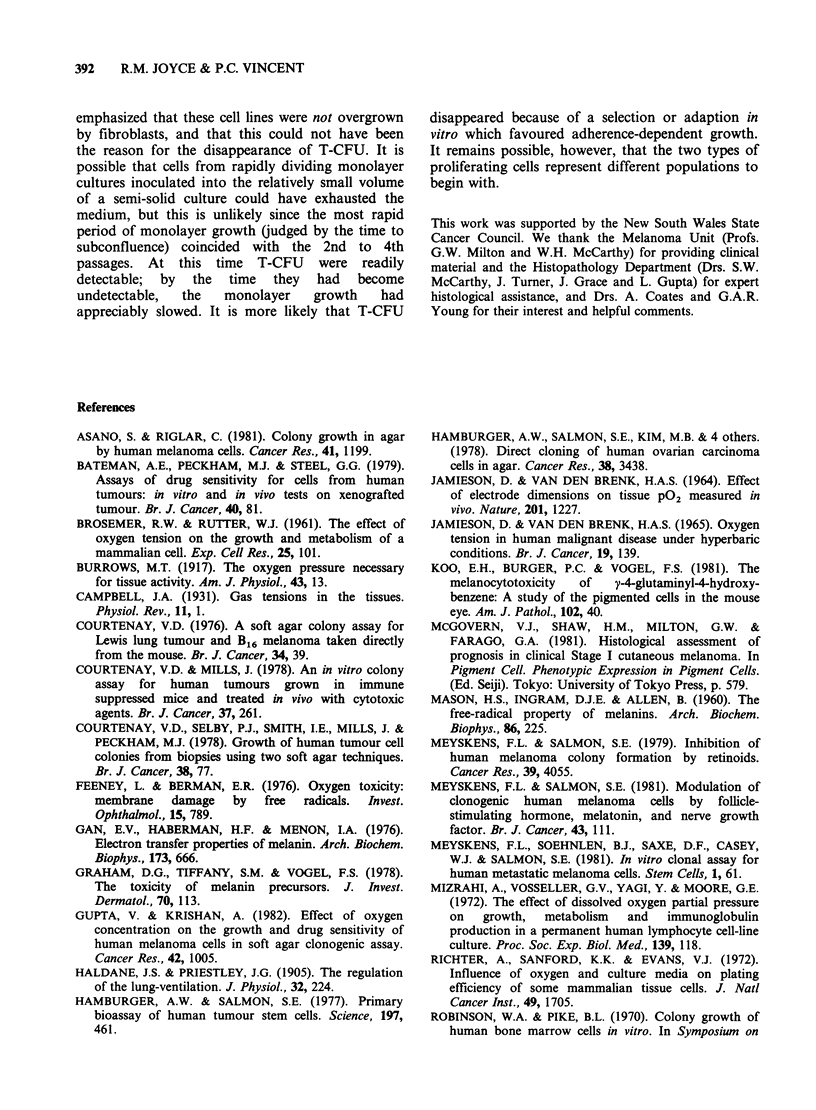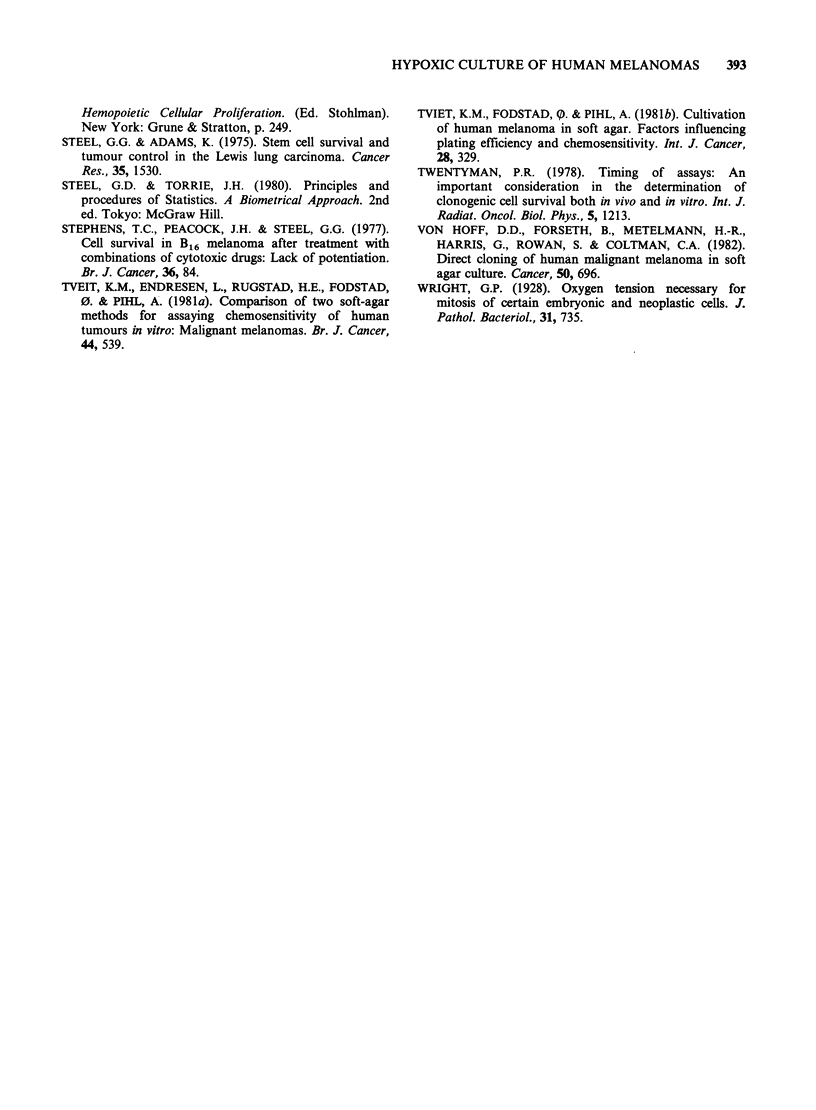# Advantage of reduced oxygen tension in growth of human melanomas in semi-solid cultures: quantitative analysis.

**DOI:** 10.1038/bjc.1983.203

**Published:** 1983-09

**Authors:** R. M. Joyce, P. C. Vincent

## Abstract

A systematic study was undertaken to compare the growth characteristics of human melanomas in liquid monolayer cultures at ambient oxygen tension, and in semi-solid cultures at ambient or reduced oxygen tension. Physically dispersed single cell suspensions from 200 freshly-excised melanomas (66 primary, 134 secondary) from 169 patients were cultured in monolayers, or plated in semi-solid cultures maintained either in 5% CO2 in room air (20% O2) or in 5% CO2, 5% O2 and 90% N2, to assay tumour colony-forming units (T-CFU). Aliquots were taken at each passage of the monolayer cultures for T-CFU assay in semi-solid culture at ambient and reduced O2 concentrations. Of 200 melanomas tested, 153 (77%) grew in monolayer culture, 94 (47%) in semi-solid culture at 5% O2, and only 48 (24%) in semi-solid culture at 20% O2. The mean number (+/-s.e.) of colonies in the 94 tumours which grew in semi-solid culture at 5% O2 (29 +/- 4 per 5 x 10(5) cells plated) was significantly greater than the mean in the same tumours in semi-solid culture at 20% O2 (11 +/- 2 per 5 x 10(5) cells). Furthermore, hypoxic colonies showed a morphologically different growth pattern. There was a significant correlation (r = 0.749, P less than 0.001) between the number of colonies growing at 5% O2 and the number at 20% O2; hypoxia appeared to act both by recruiting additional T-CFU and by increasing the proliferative activity of those already present. Short-term monolayer cultured cell lines showing evidence of persistent tumour cell characteristics were successfully established from 74 tumours, and the proportions of T-CFU assayed at each passage. In 63% of cultures the proportion of T-CFU increased initially and then declined, while in the remainder it declined progressively throughout. Although monolayer cultures were successfully maintained for up to 15 passages, T-CFU became undetectable by the eighth passage and remained so thereafter.


Br. J. Cancer (1983), 48, 385–393

### Advantage of reduced oxygen tension in growth of human melanomas in semi-solid cultures: Quantitative analysis

R.M. Joyce & P.C. Vincent

*Medical Research Department, Kanematsu Memorial Institute, Sydney Hospital, Macquarie Street, Sydney, N.S.W. 2000, Australia.*

**Summary** A systematic study was undertaken to compare the growth characteristics of human melanomas in liquid monolayer cultures at ambient oxygen tension, and in semi-solid cultures at ambient or reduced oxygen tension. Physically dispersed single cell suspensions from 200 freshly-excised melanomas (66 primary, 134 secondary) from 169 patients were cultured in monolayers, or plated in semi-solid cultures maintained either in 5% $CO_2$ in room air (20% $O_2$) or in 5% $CO_2$, 5% $O_2$ and 90% $N_2$, to assay tumour colony-forming units (T-CFU). Aliquots were taken at each passage of the monolayer cultures for T-CFU assay in semi-solid culture at ambient and reduced $O_2$ concentrations.

Of 200 melanomas tested, 153 (77%) grew in monolayer culture, 94 (47%) in semi-solid culture at 5% $O_2$, and only 48 (24%) in semi-solid culture at 20% $O_2$. The mean number ($\pm$s.e.) of colonies in the 94 tumours which grew in semi-solid culture at 5% $O_2$ ($29\pm4$ per $5\times10^5$ cells plated) was significantly greater than the mean in the same tumours in semi-solid culture at 20% $O_2$ ($11\pm2$ per $5\times10^5$ cells). Furthermore, hypoxic colonies showed a morphologically different growth pattern. There was a significant correlation ($r=0.749$, $P<0.001$) between the number of colonies growing at 5% $O_2$ and the number at 20% $O_2$; hypoxia appeared to act both by recruiting additional T-CFU and by increasing the proliferative activity of those already present.

Short-term monolayer cultured cell lines showing evidence of persistent tumour cell characteristics were successfully established from 74 tumours, and the proportions of T-CFU assayed at each passage. In 63% of cultures the proportion of T-CFU increased initially and then declined, while in the remainder it declined progressively throughout. Although monolayer cultures were successfully maintained for up to 15 passages, T-CFU became undetectable by the eighth passage and remained so thereafter.

The cells which give rise to colonies when tumours are cultured in a semi-solid medium (tumour colony-forming units, or T-CFU) are generally regarded as being tumour stem cells (Courtenay, 1976; Hamburger & Salmon, 1977; Hamburger *et al.*, 1978), although formal proof of this assumption has not yet been provided. This culture system has been extensively studied as a means of possibly determining *in vitro* the effectiveness of cytotoxic drugs (Meyskens *et al.*, 1981), but in the case of human tumours less attention has been paid to the biological behaviour of the T-CFU's themselves. One aspect of the behaviour of these cells which has however attracted considerable attention has been their enhanced growth under conditions of reduced oxygen tension, which has been shown for murine tumours (Steel & Adams, 1975; Courtenay, 1976; Stephens *et al.*, 1977), for xenografts of human tumours (Courtenay & Mills, 1978; Bateman *et al.*, 1979; Gupta & Krishan, 1982) and for freshly excised human melanomas (Courtenay *et al.*, 1978; Tveit *et al.*, 1981*a,b*). In many of these studies the addition of rat red blood cells has further enhanced colony growth (Courtenay & Mills, 1978; Tveit *et al.*, 1981*a*). In other studies, 2 mercaptoethanol has been shown to increase the cloning efficiency of human melanomas in semi-solid culture (Asano & Riglar, 1981).

With the exception of the papers by Tveit *et al.* (1981*a,b*) we can find no reports systematically comparing the growth of human melanomas under reduced or ambient oxygen tensions. Many of the larger series reported, for example, have only looked at T-CFU numbers in cultures maintained in 20% oxygen (Meyskens & Salmon, 1979, 1981; Meyskens *et al.*, 1981) or in 20% oxygen with 2 mercaptoethanol (von Hoff *et al.*, 1982). Furthermore, the overwhelming majority of studies have been carried out on metastatic melanomas, and very few data are available on the growth of human primary melanomas in culture (Courtenay *et al.*, 1978).

In this paper, we report the growth in culture of a large series (200 cases) of surgically-excised human melanomas, of which 66 were primary and 134 were metastatic. In all cases, growth in semi-solid culture under conditions of reduced oxygen ($pO_2=40$ mmHg) or ambient oxygen

Correspondence: P.C. Vincent

Received 27 April 1983; accepted 14 June 1983.

© The Macmillan Press Ltd., 1983

($pO_2 = 150$ mmHg) concentrations and in monolayer culture at ambient oxygen tension were compared. Short-term cell lines with persistent evidence of malignant cell proliferation were established from 74 tumours, and the behaviour of T-CFU in these cell lines was documented.

### Materials and methods

### *Tumours*

Sixty-six primary and 134 metastatic malignant melanomas were obtained surgically from 169 patients attending the Melanoma Unit at Sydney Hospital between June 1980 and September 1982. The tumour samples were histologically confirmed as melanoma and classified as melanotic or amelanotic, according to previously defined criteria (McGovern *et al.*, 1981). One hundred and fifty-two melanomas (51 primary, 101 metastatic) were melanotic and 48 (15 primary, 33 metastatic) were amelanotic. Tumour samples were immediately obtained for culture. Capsular and necrotic components were removed and the tumour pieces rinsed in serum-free tissue culture medium.

### *Tumour cell disaggregation*

Specimens were mechanically dissociated by mincing in the absence of medium and enzymes with fine iris scissors on a watchglass within a petri dish. Tveit *et al.* (1981*b*) have shown that mechanical disaggregation yields similar culture results to those obtained with enzyme digestion. Culture medium was added to the tumour material and drawn up and down in a Pasteur pipette several times. The resultant suspension was transferred to a 50 ml test-tube, filled with more culture medium and the larger tissue fragments allowed to settle for 10 min. The predominantly single-celled supernatant was aspirated through a 26-gauge needle into a syringe and thence to a second centrifuge tube. For each tumour, some monolayer cultures (see below) were established from this suspension, and others from the fragments which had settled after mincing. Further steps taken to ensure a single-cell suspension prior to plating of semi-solid cultures included centrifugation at 1,000 g for 7 min, removal of cell debris, resuspension in serum-free medium, and passage through a series of 26-gauge needles. A drop of this preparation was examined at $\times 200$ magnification to confirm its single cell nature and counted using a haemocytometer with phase illumination immediately prior to plating. Light, bright, intact cells were scored as viable.

### *Liquid monolayer culture*

Crude single-cell suspensions, or small fragments, were placed into separate 25 $cm^2$ or 75 $cm^2$ tissue culture flasks (Corning, New York, U.S.A.) containing a complete growth medium of Dulbecco's Modified Eagles Medium (DMEM; Gibco Laboratories, New York, U.S.A.; Flow Laboratories, Irvine, Scotland), supplemented with HEPES buffer (10 mM; Flow Laboratories, Irvine Scotland), glucose (1 mg $ml^{-1}$), sodium pyruvate (0.5 mg $ml^{-1}$) and containing penicillin (50 I.U. $ml^{-1}$), streptomycin (40 $\mu g\ ml^{-1}$) and amphotericin (2.5 $\mu g\ ml^{-1}$). Heat inactivated foetal calf serum (FCS; C.S.L., Melbourne, Australia) was added at a final concentration of 15% by volume. Flasks were incubated at 37°C in a humidified atmosphere of 5% $CO_2$ in air. Cell adherence to the plastic substrate became apparent after 12–100 h and cell outgrowth from explant material was observed within a few days. Fresh, complete growth medium and heat inactivated foetal calf serum (which was varied in the range 10–20% according to cell growth) were added to the cultures every 2–3 days. When either single-cell-type or fragment (explant)-type growth was still subconfluent and estimated to be in the upper exponential growth phase, the cells were passaged by decanting the growth medium, rinsing the monolayer cell surface twice with versene buffered saline (C.S.L.) and incubating with a large volume of Hanks balanced salt solution (Flow Laboratories) at 37°C for 10–30 min. Three or four sharp knocks brought the cells into suspension, and cells from all flasks were pooled and centrifuged at 1,000 g for 5 min. If, after 30 min, microscopic examination revealed still-adherent cells, a weak trypsin solution of 0.01% was added, but this was rarely necessary.

Monolayer cultures were serially passaged at subconfluence (Twentyman, 1978) to establish short-term cell lines and any cell lines showing evidence of overgrowth by fibroblasts were discarded. Those lines established from pigmented tumours grew as pigmented spindle cells or pigmented polygonal cells (20–35 $\mu$m in diameter) or occasionally as mixtures of the two. Lines established from non-pigmented tumours were only maintained if non-pigmented polygonal cells were present; tumours growing as non-pigmented spindle cells were presumed possibly to be fibroblastic and were discarded. In a proportion of cases flow cytometry of propidium iodide-stained cells using an Ortho System 50 H cytofluorograf with a 5 W Argon ion laser (Ortho Instruments, Westwood, Ma., U.S.A.) was employed to demonstrate persistent aneuploidy in cell lines from those tumours which were aneuploid to begin with (Wass *et al.*, in preparation).

At each passage of these short-term cell lines, single cell suspensions were prepared for plating in semi-solid agar as described above. These were designated $P_1$, $P_2 \ldots P_n$ for the first, second and subsequent passages. Using this nomenclature, semi-solid cultures derived directly from a single cell suspension of the original tumour were designated $P_0$. Cell lines were propagated for between 3 and 6 passages after the point at which T-CFU could no longer be detected.

*Semi-solid agar cultures*

A support layer of DMEM containing 15% heat inactivated foetal calf serum and 0.5% agar (Difco Laboratories, Michigan, U.S.A.) was plated at a volume of 1 ml per dish (Meyskens *et al.*, 1981) into 35 mm tissue culture dishes (Falcon Plastics, Maryland, U.S.A.), allowed to set and placed into the appropriate culture atmosphere. No conditioned medium was incorporated. Overlayers containing the cells to be tested were prepared in a similar manner with DMEM, enriched with vitamin and amino acid supplements (Robinson & Pike, 1970), insulin (preservative-free, $3.2\,u\,ml^{-1}$; C.S.L., Melbourne) and 15% heat inactivated FCS. This enriched growth medium was mixed with an appropriate volume of agar to give a final agar concentration of 0.3% and cells were added to give a final concentration of $5 \times 10^5$ cells $ml^{-1}$ as described by Meyskens *et al.* (1981) and von Hoff *et al.* (1982). A 1 ml volume was immediately plated over the support layer and allowed to gel. Dishes were inspected immediately after plating using an inverted microscope at $\times 100$ magnification, and any showing cell aggregation were discarded. As noted above, semi-solid cultures were established either from the original tumour ($P_0$) or from serial passages of monolayer cultures ($P_1$, $P_2 \ldots P_n$).

*Incubation*

Semi-solid cultures were incubated, in triplicate, for 21 days at 37°C either in a gassed incubator containing a humidified atmosphere of 5% $CO_2$ in air (150 mmHg $O_2$; Hamburger & Salmon, 1977) or in sealed vacuum dessicators gassed with a specially prepared humidified gas mixture of 5% $CO_2$, 5% $O_2$ and 90% $N_2$ (40 mmHg $O_2$; CIG, Australia; Courtenay, 1976; Courtenay & Mills, 1978). The vacuum dessicators were flushed for 10 min every 2–3 days by bubbling the 5% $CO_2$, 5% $O_2$, 90% $N_2$ gas mixture through water in the dessicator base. Control cultures in vacuum dessicators equilibrated with the 150 mmHg $O_2$ atmosphere showed no difference in growth from those maintained in 150 mmHg $O_2$ in the gassed incubator.

*Analysis of semi-solid cultures*

Dishes were examined for colony formation using an Olympus inverted microscope with grid. Colonies were designated as groups of >50 cells. (Courtenay *et al.*, 1978) and clusters as aggregates of between 6 and 50 cells, and the means of triplicate dishes were recorded.

*Statistical analysis*

Data were analysed using student's *t*-test for mean paired differences, the $\chi^2$ test corrected for continuity, or linear regression analysis, as appropriate (Steel & Torrie, 1980).

### Results

Of 200 melanomas tested in all 3 culture systems, 153 (77%) grew in primary monolayer cultures at 150 mmHg $O_2$, 94 (47%) in primary semi-solid culture at 40 mmHg $O_2$, and only 48 (24%) in primary semi-solid culture at 150 mmHg $O_2$ (Table I). Tumours which grew in monolayer culture were significantly more likely also to grow in semi-solid cultures at 40 mmHg $O_2$ ($\chi^2 = 27.137$, $P < 0.001$), and these in turn were significantly more likely to grow in semi-solid cultures at 150 mmHg $O_2$ ($\chi^2 = 68.449$, $P < 0.001$). Only 6 tumours which failed to grow in monolayer culture grew in semi-solid culture, but all of these grew at both 40 mmHg and at 150 mmHg $O_2$. Short-term cell lines satisfying the criteria for persistent malignant cell proliferation (see **Materials and methods**) were successfully established from 74/153 tumours which

**Table I** Comparison of growth of 200 human melanomas in primary culture as monolayers or in semi-solid agar at ambient or reduced oxygen tension

| | *Successful cultures obtained in*: | | | | | *Number of tumours* |
|---|---|---|---|---|---|---|
| Growth in: | | | | | | |
| Primary monolayer culture | + | + | + | − | − | 153 |
| Semi-solid agar culture: | | | | | | |
| 40 mmHg $O_2$ | + | + | − | + | − | 94 |
| 150 mmHg $O_2$ | + | − | − | + | − | 48 |
| Number of tumours: | 42 | 46 | 65 | 6 | 41 | 200 |

+: Growth.
−: Failure to grow.
Association between growth in monolayer and in semi-solid cultures at 40 mmHg $O_2$; $\chi^2$ (1 d.f.) = 27.137, $P < 0.001$.
Association between growth in monolayer and in semi-solid cultures at 150 mmHg $O_2$; $\chi^2$ (1 d.f.) = 3.484, $0.1 > P > 0.05$.
Association between growth in semi-solid cultures at 40 mmHg $O_2$ and at 150 mmHg $O_2$; $\chi^2$ (1 d.f.) = 68.449, $P < 0.001$.

initially grew in monolayer culture, and were maintained for up to 15 passages over 24 weeks.

Details of the growth characteristics in the 3 culture systems for primary or metastatic, melanotic or amelanotic tumours are shown in Table II. Metastatic melanomas grew more frequently than primary melanomas in liquid monolayer cultures (109/134 *vs.* 44/66, $\chi^2=4.513$, $0.05>P>0.01$), but there was no significant difference in growth between metastatic and primary tumours in semi-solid cultures, either at 40 mmHg $O_2$ or at 150 mmHg $O_2$. The greater likelihood of growth in monolayer culture than in semi-solid cultures at 40 mmHg $O_2$ held true for all tumour sub-types except for primary amelanotic melanomas which however was a small sample (only 15 cases) (Table II, a *vs.* b). All tumour sub-types, except primary amelanotic melanomas, showed a significantly higher incidence of successful growth in semi-solid culture at 40 mmHg $O_2$, compared with 150 mmHg $O_2$ (Table II, b *vs.* c). Growth characteristics for melanotic and amelanotic tumours were not significantly different from each other in any of the 3 culture systems. The significantly more frequent growth of tumours in primary monolayer culture than in semi-solid culture at 40 mmHg $O_2$, in which in turn the success rate was higher than at 150 mmHg $O_2$ (Table I) was also apparent when primary or metastatic tumours, or melanotic tumours were analysed separately (Table II).

The mean number of tumour-colony forming units (T-CFU) in the 94 tumours which grew in semi-solid culture at 40 mmHg $O_2$ was $29\pm4$ per $5\times10^5$ cells plated (mean $\pm$s.e.). Only 48 of these tumours grew at 150 mmHg $O_2$, with a significantly lower mean ($\pm$s.e.) colony count of $11\pm2$ per $5\times10^5$ cells (mean paired difference; $t=6.81$, $P<0.001$).

The linear regression between the number of colonies grown at 40 mmHg $O_2$ ($y$) and at 150 mmHg $O_2$ ($x$) for each tumour is shown in Figure 1. The relationship ($y=1.32\ x+14.6$) shows a highly significant correlation ($r=0.749$, $P<0.001$), with a slope (1.32) which is significantly greater ($0.02>P>0.01$) than unity and a $y$ intercept (14.6) which differs significantly ($P<0.001$) from zero. These results suggest that hypoxia increases colony growth due to two effects; first, an additional, more or less fixed, number of T-CFU are induced to divide, displacing the relationship upwards (indicated by the significant $y$ intercept value), and second, for each unit increase in the number of T-CFU grown under ambient conditions the number grown under hypoxic conditions increases by 1.32 (indicated by the slope of the line).

As with freshly-obtained material, the success rate and plating efficiency of T-CFU cultures from each of the 74 short-term cell lines studied were greater with cultures incubated in reduced (40 mmHg) $O_2$ conditions. In hypoxic semi-solid cultures from 47 short-term cell lines (63%), the proportion of T-CFU increased for the first 1, 2 or 3 passages and then declined (12 representative experiments of this type are shown in the upper panel of Figure 2). In similar cultures from the remaining 27 melanomas (37%) the proportion of T-CFU declined

**Table II** Growth in 3 culture systems of 200 melanomas, classified according to primary or metastatic origin and histological type

| | | *Numbers of each tumour sub-type growing:* | | | | |
|---|---|---|---|---|---|---|
| | | *(a)* *In monolayer culture* *(150 mmHg $O_2$)* | *(b)* *In semi-solid culture* *(40 mmHg $O_2$)* | *(c)* *In semi-solid culture* *(150 mmHg $O_2$)* | *Significance* *a* vs. *b* | *b* vs. *c* |
| Primary melanomas: | | | | | | |
| Melanotic | (51) | 37 (72.5%) | 21 (41.2%) | 7 (13.7%) | ** | ** |
| Amelanotic | (15) | 7 (46.7%) | 6 (40.0%) | 5 (33.3%) | NS | NS |
| All primary | (66) | 44 (66.7%) | 27 (40.9%) | 12 (18.2%) | ** | ** |
| Metastatic melanomas: | | | | | | |
| Melanotic | (101) | 83 (82.2%) | 48 (47.5%) | 28 (27.7%) | *** | ** |
| Amelanotic | (33) | 26 (78.8%) | 19 (57.6%) | 8 (24.2%) | NS | * |
| All metastatic | (134) | 109 (81.3%) | 67 (50.0%) | 36 (26.9%) | *** | *** |
| Total | (200) | 153 (76.5%) | 94 (47.0%) | 48 (24.0%) | *** | *** |
| Significance, primary *vs.* metastatic | | * | NS | NS | | |

*$0.05>P>0.01$.
**$0.01>P>0.001$.
***$P<0.001$.
NS Not significant.
Analyses using $\chi^2$ with 1 d.f.

**Figure 1** Comparison of the growth of human melanomas in semi-solid culture at reduced ($pO_2$ = 40 mmHg) or ambient ($pO_2$ = 150 mmHg) oxygen tensions. Results are shown only for those tumours (94 samples) where growth occurred in one or other culture system. Each point represents the paired results of triplicate cultures of one tumour at each oxygen tension. The 95% confidence intervals for the regression line are shown by dotted lines.

progressively from the original ($P_0$) value (12 representative results shown in the lower panel, Figure 2). In every case, semi-solid cultures at 150 mmHg $O_2$ gave parallel results. All cell lines tested eventually reached a point where semi-solid culture failed to yield colonies, although increased numbers (2–3 fold) of single cells and small (2–4 cell) clusters were still present. This pattern persisted for the remaining life of all the lines tested, despite the continued ability of the cells to grow vigorously in monolayer cultures.

### Discussion

The ability of tumour cells to form colonies in semi-solid culture has become widely accepted as a means of evaluating the behaviour and chemotherapeutic sensitivity of tumours (Hamburger & Salmon, 1977). Intrinsic in this assumption is the concept that the proportion of cells capable of forming colonies—i.e., the plating efficiency—represents the stem cell population in the tumour. However this concept is hard to reconcile with

**Figure 2** Representative graphs showing the proportions of T-CFU (grown at $pO_2 = 40$ mmHg) from human melanomas propagated in monolayer culture. Of 74 melanomas serially studies until T-CFU could no longer be detected, 47 (63%) showed an initial increase in numbers of T-CFU (results from 12 cultures shown in upper panel) and 27 (37%) showed a progressive decline (results from 12 cultures shown in lower panel). Semi-solid cultures at $pO_2$ 150 mmHg gave parallel results with uniformly lower numbers of T-CFU (not shown).

observations that the plating efficiency can be increased by changing the culture conditions, such as by the use of reduced oxygen tension and/or adding rat erythrocytes (Steel & Adams, 1975; Courtenay, 1976; Courtenay & Mills, 1978; Courtenay *et al.*, 1978; Tveit *et al.*, 1981*a, b*; Gupta & Krishan, 1982), or of 2 mercaptoethanol (Asano & Riglar, 1981; von Hoff *et al.*, 1982) or trophic hormones (Meyskens & Salmon, 1981). With such variations possible in the proportions of tumour cells capable of forming colonies in culture, it is difficult to be sure how large the stem cell population really is or whether conventional cultures adequately assay its behaviour.

Our data from 200 human melanomas cultured directly in semi-solid agar confirm, in a large series, the greater proportion of successful "takes", and the higher plating efficiency, in semi-solid cultures maintained at reduced oxygen tensions. Furthermore, regression analysis comparing paired results from each tumour indicates that hypoxia recruits an additional number of cells capable of forming colonies *and* increases the numbers of colonies any given tumour will form. It has yet to be established whether the additional colony-forming cells in hypoxic cultures come from the same progenitor pool as those growing in ambient $O_2$, or whether they represent a separate population. In practical terms, however, the results suggest that semi-solid cultures, at least from melanomas, might best be performed under hypoxic conditions.

It is not clear whether higher oxygen tensions are toxic or lower ones stimulatory. It has been known for many years that oxygen tension in tissues is much less than in air (Haldane & Priestley, 1905), and Campbell (1931) found that oxygen tension was 20–40 mmHg in all tissues except brain and lung. The advantages of culturing tissues under hypoxic conditions were first recognized by Burrows (1917) and have been confirmed extensively since (Wright, 1928; Brosemer & Rutter, 1961; Jamieson & van den Brenck, 1964, 1965; Mizrahi *et al.*, 1972). Ritchter *et al.* (1972) showed that plating efficiencies of monolayer culture-grown cells were appreciably higher under low oxygen tensions for both neoplastic and most non-neoplastic cells. Feeney & Berman (1976) suggested that transient free radicals produced in the presence of oxygen might irreversibly damage enzymes and membrane lipids, and Brosemer & Rutter (1961) found that high oxygen tensions inhibited cellular proliferation. Graham *et al.* (1978) identified dopaquinone, a highly reactive intermediate resulting from tyrosine-mediated oxidation, as a significant toxic metabolite in melanin biosynthesis. Melanin itself can function as a biological electron transfer agent with remarkable specificities in its oxidizing properties (Mason *et al.*, 1960; Gan *et al.*, 1976; A. Coates, personal communication) and this could contribute to oxygen toxicity in an active melanogenic environment (Koo *et al.*, 1981).

Tumour cells can be cultured either as monolayers, where growth is dependent on cellular adherence to the plastic, or as semi-solid agar cultures, or liquid suspension cultures, where growth is independent of adherence. It is not clear however whether the same population of cells is responsible for growth under each of these conditions (i.e., adherence-dependent or adherence-independent growth), or whether two distinct populations are responsible. Asano & Riglar (1981) compared the growth characteristics of melanoma cell lines in monolayer and in semi-solid culture, but we can find no report of similar comparisons being made using freshly-obtained samples of human melanoma.

To answer this question, we have compared directly the growth of biopsy-obtained melanomas in both monolayer and semi-solid culture systems, and the growth in semi-solid culture of cell lines derived from the same tumours. Melanomas obtained directly from biopsies grew significantly more frequently in monolayer culture than in semi-solid cultures even when the latter were incubated under hypoxic conditions. However, the great majority (94%) of successful semi-solid cultures came from tumours which also grew in primary monolayer culture.

The behaviour of cells capable of adherence-independent growth—i.e., T-CFU—in the short-term cell lines established from these melanomas was studied by serially sampling for T-CFU numbers at each passage of the culture. In 64% of the short-term cell lines, the proportions of T-CFU increased in the 1st, 2nd or 3rd passage, while in the remainder the proportions declined progressively throughout. The initial rise in the proportions of T-CFU in the majority of tumours is intriguing, and could represent either (1) removal of *in vivo* inhibitory factors by passage, (2) the recruitment of quiescent ($G_0$) cells into cycle over the first few passages in monolayer culture, or (3) transient latency while the cells adapt to *in vitro* growth. Whatever the reason, these studies indicate that primary semi-solid cultures could be misleading if the true proportions of T-CFU do not become apparent until several passages through monolayer culture, and suggest that the clonogenic assay may measure an unrepresentative subpopulation of the potentially proliferative cells in a tumour.

T-CFU ultimately disappeared from all short-term cell lines, despite the continued ability of these cultures to grow vigorously as monolayers with morphological and flow cytometric evidence of persistent tumour cell characteristics. It must be emphasized that these cell lines were *not* overgrown by fibroblasts, and that this could not have been the reason for the disappearance of T-CFU. It is possible that cells from rapidly dividing monolayer cultures inoculated into the relatively small volume of a semi-solid culture could have exhausted the medium, but this is unlikely since the most rapid period of monolayer growth (judged by the time to subconfluence) coincided with the 2nd to 4th passages. At this time T-CFU were readily detectable; by the time they had become undetectable, the monolayer growth had appreciably slowed. It is more likely that T-CFU disappeared because of a selection or adaption *in vitro* which favoured adherence-dependent growth. It remains possible, however, that the two types of proliferating cells represent different populations to begin with.

This work was supported by the New South Wales State Cancer Council. We thank the Melanoma Unit (Profs. G.W. Milton and W.H. McCarthy) for providing clinical material and the Histopathology Department (Drs. S.W. McCarthy, J. Turner, J. Grace and L. Gupta) for expert histological assistance, and Drs. A. Coates and G.A.R. Young for their interest and helpful comments.

### References

ASANO, S. & RIGLAR, C. (1981). Colony growth in agar by human melanoma cells. *Cancer Res.*, **41**, 1199.

BATEMAN, A.E., PECKHAM, M.J. & STEEL, G.G. (1979). Assays of drug sensitivity for cells from human tumours: *in vitro* and *in vivo* tests on xenografted tumour. *Br. J. Cancer*, **40**, 81.

BROSEMER, R.W. & RUTTER, W.J. (1961). The effect of oxygen tension on the growth and metabolism of a mammalian cell. *Exp. Cell Res.*, **25**, 101.

BURROWS, M.T. (1917). The oxygen pressure necessary for tissue activity. *Am. J. Physiol.*, **43**, 13.

CAMPBELL, J.A. (1931). Gas tensions in the tissues. *Physiol. Rev.*, **11**, 1.

COURTENAY, V.D. (1976). A soft agar colony assay for Lewis lung tumour and $B_{16}$ melanoma taken directly from the mouse. *Br. J. Cancer*, **34**, 39.

COURTENAY, V.D. & MILLS, J. (1978). An *in vitro* colony assay for human tumours grown in immune suppressed mice and treated *in vivo* with cytotoxic agents. *Br. J. Cancer*, **37**, 261.

COURTENAY, V.D., SELBY, P.J., SMITH, I.E., MILLS, J. & PECKHAM, M.J. (1978). Growth of human tumour cell colonies from biopsies using two soft agar techniques. *Br. J. Cancer*, **38**, 77.

FEENEY, L. & BERMAN, E.R. (1976). Oxygen toxicity: membrane damage by free radicals. *Invest. Ophthalmol.*, **15**, 789.

GAN, E.V., HABERMAN, H.F. & MENON, I.A. (1976). Electron transfer properties of melanin. *Arch. Biochem. Biophys.*, **173**, 666.

GRAHAM, D.G., TIFFANY, S.M. & VOGEL, F.S. (1978). The toxicity of melanin precursors. *J. Invest. Dermatol.*, **70**, 113.

GUPTA, V. & KRISHAN, A. (1982). Effect of oxygen concentration on the growth and drug sensitivity of human melanoma cells in soft agar clonogenic assay. *Cancer Res.*, **42**, 1005.

HALDANE, J.S. & PRIESTLEY, J.G. (1905). The regulation of the lung-ventilation. *J. Physiol.*, **32**, 224.

HAMBURGER, A.W. & SALMON, S.E. (1977). Primary bioassay of human tumour stem cells. *Science*, **197**, 461.

HAMBURGER, A.W., SALMON, S.E., KIM, M.B. & 4 others. (1978). Direct cloning of human ovarian carcinoma cells in agar. *Cancer Res.*, **38**, 3438.

JAMIESON, D. & VAN DEN BRENK, H.A.S. (1964). Effect of electrode dimensions on tissue $pO_2$ measured *in vivo*. *Nature*, **201**, 1227.

JAMIESON, D. & VAN DEN BRENK, H.A.S. (1965). Oxygen tension in human malignant disease under hyperbaric conditions. *Br. J. Cancer*, **19**, 139.

KOO, E.H., BURGER, P.C. & VOGEL, F.S. (1981). The melanocytotoxicity of γ-4-glutaminyl-4-hydroxybenzene: A study of the pigmented cells in the mouse eye. *Am. J. Pathol.*, **102**, 40.

MCGOVERN, V.J., SHAW, H.M., MILTON, G.W. & FARAGO, G.A. (1981). Histological assessment of prognosis in clinical Stage I cutaneous melanoma. In *Pigment Cell. Phenotypic Expression in Pigment Cells.* (Ed. Seiji). Tokyo: University of Tokyo Press, p. 579.

MASON, H.S., INGRAM, D.J.E. & ALLEN, B. (1960). The free-radical property of melanins. *Arch. Biochem. Biophys.*, **86**, 225.

MEYSKENS, F.L. & SALMON, S.E. (1979). Inhibition of human melanoma colony formation by retinoids. *Cancer Res.*, **39**, 4055.

MEYSKENS, F.L. & SALMON, S.E. (1981). Modulation of clonogenic human melanoma cells by follicle-stimulating hormone, melatonin, and nerve growth factor. *Br. J. Cancer*, **43**, 111.

MEYSKENS, F.L., SOEHNLEN, B.J., SAXE, D.F., CASEY, W.J. & SALMON, S.E. (1981). *In vitro* clonal assay for human metastatic melanoma cells. *Stem Cells*, **1**, 61.

MIZRAHI, A., VOSSELLER, G.V., YAGI, Y. & MOORE, G.E. (1972). The effect of dissolved oxygen partial pressure on growth, metabolism and immunoglobulin production in a permanent human lymphocyte cell-line culture. *Proc. Soc. Exp. Biol. Med.*, **139**, 118.

RICHTER, A., SANFORD, K.K. & EVANS, V.J. (1972). Influence of oxygen and culture media on plating efficiency of some mammalian tissue cells. *J. Natl Cancer Inst.*, **49**, 1705.

ROBINSON, W.A. & PIKE, B.L. (1970). Colony growth of human bone marrow cells *in vitro*. In *Symposium on*

*Hemopoietic Cellular Proliferation.* (Ed. Stohlman). New York: Grune & Stratton, p. 249.

STEEL, G.G. & ADAMS, K. (1975). Stem cell survival and tumour control in the Lewis lung carcinoma. *Cancer Res.*, **35**, 1530.

STEEL, G.D. & TORRIE, J.H. (1980). Principles and procedures of Statistics. *A Biometrical Approach.* 2nd ed. Tokyo: McGraw Hill.

STEPHENS, T.C., PEACOCK, J.H. & STEEL, G.G. (1977). Cell survival in $B_{16}$ melanoma after treatment with combinations of cytotoxic drugs: Lack of potentiation. *Br. J. Cancer*, **36**, 84.

TVEIT, K.M., ENDRESEN, L., RUGSTAD, H.E., FODSTAD, Ø. & PIHL, A. (1981*a*). Comparison of two soft-agar methods for assaying chemosensitivity of human tumours *in vitro*: Malignant melanomas. *Br. J. Cancer*, **44**, 539.

TVIET, K.M., FODSTAD, Ø. & PIHL, A. (1981*b*). Cultivation of human melanoma in soft agar. Factors influencing plating efficiency and chemosensitivity. *Int. J. Cancer*, **28**, 329.

TWENTYMAN, P.R. (1978). Timing of assays: An important consideration in the determination of clonogenic cell survival both *in vivo* and *in vitro*. *Int. J. Radiat. Oncol. Biol. Phys.*, **5**, 1213.

VON HOFF, D.D., FORSETH, B., METELMANN, H.-R., HARRIS, G., ROWAN, S. & COLTMAN, C.A. (1982). Direct cloning of human malignant melanoma in soft agar culture. *Cancer*, **50**, 696.

WRIGHT, G.P. (1928). Oxygen tension necessary for mitosis of certain embryonic and neoplastic cells. *J. Pathol. Bacteriol.*, **31**, 735.